# The relationship between the development of social competence and sleep in infants: a longitudinal study

**DOI:** 10.1186/s13034-018-0258-8

**Published:** 2018-12-18

**Authors:** Etsuko Tomisaki, Emiko Tanaka, Taeko Watanabe, Ryoji Shinohara, Maki Hirano, Yoko Onda, Yukiko Mochizuki, Yuko Yato, Noriko Yamakawa, Tokie Anme, the Japan Children’s Study Group

**Affiliations:** 1 Keio University, Tokyo, Japan; 2 Graduate School of Comprehensive Human Sciences, University of Tsukuba, 1-1-1 Tennodai, Tsukuba-shi, Ibaraki-ken 305-8577 Japan; 3 Shukutoku University, Chiba, Japan; 4 Health Science University, Yamanashi, Japan; 5 College of Letters, Ritsumeikan University, Kyoto, Japan; 6 Clinical Research Institute, Mie-Chuo Medical Center, National Hospital Organization, Tsu, Japan

**Keywords:** Social competence, Nighttime sleep duration, Total sleep duration, Sleep onset time, Longitudinal study

## Abstract

**Background:**

Many reports argue that sleep is important for children’s health, learning, and academic performance. The purpose of this longitudinal study was to examine the association between sleep and the development of social competence in infants.

**Methods:**

This study was conducted as part of a Japan Science and Technology Agency (JST) project. Caregivers responded to the Japan Children’s Study Sleep Questionnaire when children were 18 months old. The interactions of caregivers and children were observed when children were 18, 30, and 42 months old, and rated with the Interaction Rating Scale, which is a measure of social competence.

**Results:**

Nocturnal sleep duration of more than 10 h and an earlier bed time than 22:00 were significantly correlated with two trajectory groups (low point and high point transition groups) of children’s social competence at 18, 30, and 42 months. Further, total sleep duration of more than 12.25 h and an earlier bed time than 22:00 were significantly correlated with the trajectory of children’s social competence at 18, 30, and 42 months.

**Conclusions:**

Sleep duration and sleep onset time are important factors in children’s development of social competence.

*Trial registration* The ethics committee of the JST approved this study on March 19, 2001. The registration number is 356-1.

## Background

Social competence is an ability to take another’s perspective, learn from experiences, and apply these abilities to the ever-changing social landscape [1]. Evidence links social competence to education, employment, criminal activity, substance use, and mental and physical health [2, 3]; additionally, high social competence is valued by organizations and employers, and promotes success in jobs [4]. Around 18 months, children recognize themselves in a mirror [5]. Further, they show empathy [6] and engage in cooperative interactions with others [7]. For the development of these abilities, children must recognize that they themselves and others may possess different perspectives [8]. To gain this recognition, interactions that occur with caregivers are important [9]. Social competence is receiving an increasing amount of attention in Japan, partially due to rising awareness of problems with bullying and *hikikomori* (children who are not sick but still cannot go to school because of reasons such as bullying, being unable to understand what teachers say, and loneliness in class; they stay home most of the time without any contact with society). Some studies have reported that deficits in social skills predict depressive symptoms and peer victimization [10, 11]. Others have reported that problem groups have lower social competence in elementary school [12]. From these reports, it can be said that social competence is an important factor for bullying and *hikikomori*.

Optimal sleep is known to be essential to normal growth and development, as well as to emotional health and proper immune system functioning [13, 14]. Further, sleep is critical to brain and body development [15–17]. Inadequate sleep can adversely affect all aspects of a child’s biopsychosocial health [18]. Many reports have examined the link between sleep and behavioral problems [19–24] in children. Dahl reported that inadequate sleep results in tiredness, lack of attentional focus, low negative affect thresholds (irritability and rapid frustration), and difficulty in moderating impulses and emotions [20]. Furthermore, inadequate or insufficient sleep is related to behavioral and emotional regulation [25–27], which are among the factors of social competence.

In the study of sleep, researchers must consider many factors, such as “waking up time” (the time a child wakes up), “sleep onset time” (the time a child goes to sleep), “daytime nap duration” (the total time a child sleeps during a nap), “nocturnal sleep duration” (the total time a child sleeps during the night), and “total sleep duration” (the total time a child sleeps including nap time). Further, sleep rhythm (whether sleep onset time and waking up time are consistent during a week) is also important. These factors have accordingly received considerable attention in the literature. In this study, we looked at three factors: “sleep onset time”, “nocturnal sleep duration” and “total sleep duration”.

“Late bedtimes” are correlated with problematic behaviors [28–38]. “Eveningness” has been correlated with scores on a composite measure of antisocial behavior, rule-breaking, attention behavior problems, and conduct disorder symptoms in boys, and to relational aggression in girls [39]. Late sleep onset time has been correlated with irritation in junior high students [39, 40]. Additionally, late sleep onset time has been correlated with aggressive behavior [22, 41] and the development of verbal impairments [42] in infants.

Short nocturnal sleep duration has been negatively correlated with approachability and positively correlated with hyperactivity–impulsivity [21, 43, 44], while increased nocturnal sleep has been correlated with increased approachability at 3, 6, and 11 months [44]. Further, children aged less than 3.5 years with short nocturnal sleep durations showed an increased risk of high hyperactivity–impulsivity scores and low cognitive performance at 6 years compared with children who slept 11 h per night, after controlling for potentially confounding variables [21]. Furthermore, 3 to 5-year-old children had significant correlations between sleep duration and social engagement [25].

Total sleep duration was associated with emotional problems [45, 46]. Furthermore, a shorter daytime sleep duration was correlated with emotional regulation at 12 months of age [44]; late bedtimes and less total sleeping time appear to be associated with and predictive of social-emotional problems in infants and toddlers [38].

As stated, sleep is important. Unfortunately, Japanese children had the shortest total sleeping time in a sample of 18 countries, with an average of 11.6 h per day from birth to 36 months [47]. Further, the bedtimes of Japanese children are reported to be late [48–50], and late bedtimes are specifically associated with shorter nocturnal sleep durations [51]. Late bedtime and short nocturnal and total sleep duration at a young age may strongly affect many aspects of development. For these reasons, we examined the association of sleep with social competence.

Although large, the literature on sleep contains few studies that have examined sleep’s association with social competence. The purpose of this longitudinal study was to examine the association between sleeping and the development of social competence in infants. We hypothesized that children with late bedtimes and short nocturnal and total sleep duration may also have low social competence scores.

## Methods

### Participants

Participants were drawn from the Japan Science and Technology Agency (JST) project, which operated in two cities in Japan (Osaka and Mie) from 2003 to 2009. Four hundred and sixty-five caregiver-child dyads participated in the JST project; we analyzed participants in its observation component. Children in caregiver-child dyads were aged 18 months (206 dyads), 30 months (305 dyads), and 42 months (158 dyads). Regarding trajectory of social competence, dyads who answered the paper at 18 months and participated at least twice in the observation component at 18 months, 30 months, and 42 months (207 dyads) were analyzed. We conducted a one-way ANOVA between these groups (i.e., 18-, 30-, and 42-month dyads). No significant differences were found between these groups regarding gender (F = 0.01, P = 0.94) or presence of siblings (F < 0.01, P = 0.97). Dyads were observed at 18, 30, and 42 months; social competence was rated using the Interaction Rating Scale (IRS).

### Measures

Caregivers were asked to record a daily sleep log on each day of the week regarding sleep/wake status on the Japan Children’s Study Sleep Questionnaire (JCSSQ). This measure’s reliability and validity on weekdays have been supported [52, 53]. Sleep/wake status was recorded in the daily sleep log, including sleep onset time, morning waking time, and sleep period, which were the variables extracted for analysis.

The Index of Child Care Environment (ICCE) is based on the Home Observation for Measurement of the Environment (HOME) scale [54]. This measure’s reliability and validity have been supported [55–57]. The ICCE is a screening questionnaire used to evaluate the quality of a childcare environment. It contains 13 items in four subscales: (1) human stimulation (i.e., work together with your partner to raise your child), (2) avoidance of restriction (i.e., number of times in a week you slap your child), (3) social stimulation (i.e., go to the park with your child), and (4) social support (i.e., have child care support). Some items are rated on a five-point Likert scale (from 1 to 5); others require a simple yes-or-no response.

The Interaction Rating Scale (IRS) is used in a controlled laboratory environment to obtain a rating of a child’s social competence, based on observations of the caregiver–child interaction. We have conducted observations at 18, 30, and 42 months. Trained evaluators whose concordance rate was more than 90% evaluated mother–child interactions in videos. This measure’s reliability and validity have been supported [58–60]. The IRS includes 70 behavioral and 11 impression score items in 10 subscales. Five subscales examine the child’s social competence: (1) autonomy, (2) responsiveness, (3) empathy, (4) motor regulation, and (5) emotion regulation. The other five subscales assess the caregiver’s parenting skills: (6) respect for autonomous development, (7) respect for responsiveness development, (8) respect for empathy development, (9) respect for cognitive development, and (10) respect for socio-emotional development. One item assesses the relationship’s overall synchronicity. Each subscale assesses the presence of behavior (1 = *Yes*, 0 = *No*). The IRS checklist, composed of 25 items examining the behavior of the child toward the caregivers (e.g., child looks at the caregiver’s face as a social reference) and 45 items examining the conduct of the caregiver, was completed by an observer. The total score for each child is the sum of subscale scores (maximum = 25). A higher score indicates a higher level of social competence. We used only the child’s social competence subscales in this study.

### Procedure

Primary caregivers (mostly mothers: 97.1%) provided demographic data and completed the ICCE and the JCSSQ at 18 months (Tables 1, 2) and posted them.

**Table 1:** Demographic information at 18 months

Items	18 months (n = 207)	
	n	%
Genders		
Boys	102	49.3
Girls	105	50.7
Siblings		
0	103	49.8
1	80	38.6
2	21	10.1
3	1	0.5
No answer	2	1.0
Family type		
Nuclear family	177	85.5
Extendend family	28	13.5
No answer	2	1.0
Mother’s age		
20–29	54	26.1
30–39	143	69.1
40–49	10	4.8
No answer	–	–
Father’s age		
20–29	36	17.4
30–39	138	66.7
40–49	24	11.6
50–	1	0.5
No answer	8	3.9
Mother’s career		
No	67	32.4
Yes	114	55.1
No answer	26	12.6
Family annual income		
< 2 million JPY	34	16.4
2–4 million JPY	46	22.2
4–6 million JPY	85	41.1
6–8 million JPY	26	12.6
8–10 million JPY	9	4.4
≥ 10 million JPY	7	3.4
No answer	–	–
Using child care center		
No	78	37.7
Yes	125	60.4
No answer	4	1.9

*JPY* Japanese yen

**Table 2:** The distribution of sleep at 18 months

Items	18 months (n = 207)	
	n	%
Waking up time		
5 ~ o’clock	1	0.5
6 ~ o’clock	54	26.1
7 ~ o’clock	110	53.1
8 ~ o’clock	31	15.0
9 ~ o’clock	8	3.9
10 ~ o’clock	1	0.5
11 ~ o’clock	2	1.0
Sleep onset time		
19 ~ o’clock	2	1.0
20 ~ o’clock	28	13.5
21 ~ o’clock	102	49.3
22 ~ o’clock	56	27.1
23 ~ o’clock	14	6.8
24 ~ o’clock	5	2.4
Daytime naps duration (h)		
0	12	5.8
~ 1	20	9.7
~ 2	118	57.0
~ 3	47	22.7
~ 4	10	4.8
4~	–	–
Nocturnal sleeping duration (h)		
5~	3	1.4
7~	1	0.5
8~	15	7.2
9~	98	47.3
10~	76	36.7
11~	12	5.8
12~	2	1.0
Total sleeping duration (h)		
8~	1	0.5
9~	11	5.3
10~	30	14.5
11~	79	38.2
12~	69	33.3
13~	14	6.8
14~	3	1.4

In observation, caregiver–child interactions were recorded using five video cameras (one at each of the four corners of the room and one in the center of the ceiling). Recordings were made when the children were 18, 30, and 42 months old. Each dyad was escorted to a playroom (4 × 4 m) furnished with a small table and chairs for the caregiver and child. We asked each caregiver to teach his or her child a prescribed task, which was slightly difficult for the child to accomplish alone (building a small house with some building blocks). During the house-building task, the caregiver gave instructions to and assisted the child, as in daily life. We considered that the task began when the caregiver received the building blocks and ended upon the completion of the house and the caregiver’s tidying up of the play area. Observation typically lasted for 1–5 min. An observer then completed the IRS checklist based on the video recordings of interactions.

### Analysis

The Statistical Analysis System (SAS; v9.3) was used for all data analysis. We performed Spearman rank-order correlations between the 18-month-olds’ sleep ratings (nocturnal sleep duration, total sleep duration, and sleep onset time) and social competence at 18, 30, and 42 months.

A trajectory of social competence growth was developed for each child using the semi-parametric group-based trajectory method (Proc Traj, an extension of SAS for Windows, v.9.1; SAS Institute, Inc., Cary, North Carolina) [61–63]. The selection of the optimal number of trajectory groups was based on the Bayesian information criteria (BIC). In trajectory analysis, subjects with some missing longitudinal variables were included in the analysis. The base model assumed the missing data to be random. The missing data in this research do not depend on the data value, meaning the missing data are random [64], so the base model fits the data well. Maximum likelihood estimation was used to estimate the model parameters.

After establishing the trajectories of social competence from 18 to 42 months old, the model computed the effect of predictor variables on the probability of trajectory group membership. We checked the probability of each variable in terms of which trajectory group it may belong to. A logistic regression analysis was used.

Next, we examined the contributions of a group of nocturnal sleep duration and sleep onset time, and another of total sleep duration and sleep onset time in distinguishing group memberships for child social competence trajectories. Group memberships were made at the cutoff point of 25th percentile. A multinomial logistic regression function was used.

## Results

Demographic data are shown in Table 1. Two hundred and seven caregiver-child dyads were analyzed regarding the trajectory of children’s social competence. The percentage of only children at 18 months was 49.8%, and 85.5% lived in a nuclear family.

Table 2 shows the distribution of sleep variables (i.e., waking up time, sleep onset time, daytime nap duration, nocturnal sleep duration, total sleep duration) at 18 months of age. The mean (standard deviation: SD) sleep onset time was 21.56 (0.89); 49.3% of children went to sleep between 21:00 and 22:00. The mean nocturnal sleep duration was 9.64 (0.85) hours; 47.3% of children slept from 9 to 10 h. The mean total sleep duration was 11.56 (1.02) hours per day; 38.2% slept from 11 to 12 h in total. Sleep onset time was found to be positively correlated with nocturnal sleep duration (*r* = 0.43, *P* < 0.001) and total sleep duration (r = 0.28, *P* < 0.001).

The mean score on the IRS was 21.35 (3.63) at 18 months, 22.32 (3.02) at 30 months, and 22.96 (2.66) at 42 months. Table 3 shows correlations between nocturnal sleep duration, total sleep duration, and sleep onset time and social competence. Nocturnal sleep duration was significantly positively correlated with emotion regulation scores (*r* = 0.18, *P* = 0.01) at 18 months. At 30 months, sleep onset time was significantly positively correlated with emotion regulation (*r* = 0.12, *P* = 0.04). At 42 months, sleep onset time was significantly positively correlated with emotion regulation (*r* = 0.18, *P* = 0.02), motor regulation (*r* = 0.26, *P* < 0.01), and total social competence (*r* = 0.23, *P* < 0.01). Nocturnal sleep duration was positively correlated with emotion regulation (*r* = 0.17, *P* = 0.03) and total social competence (*r* = 0.18, *P* = 0.02). Further, total sleep duration was positively correlated with total social competence (*r* = 0.18, *P* = 0.02).

**Table 3:** Correlations between sleep and child’s social competence

	Social competence		Autonomy		Responsiveness		Empathy		Motor regulation		Emotional regulation	
	r	P	r	P	r	P	r	P	r	P	r	P
Sleep onset time (18 months)	0.07	0.30	0.02	0.83	− 0.03	0.67	0.11	0.10	− 0.06	0.43	0.11	0.10
Nocturnal sleeping duration (18 months)	0.08	0.24	0.05	0.49	− 0.04	0.59	0.06	0.42	0.01	0.91	0.18	*0.01*
Total sleeping duration (18 months)	0.02	0.76	0.05	0.45	− 0.02	0.80	0.03	0.64	− 0.12	0.08	0.06	0.41
Sleep onset time (30 months)	0.08	0.14	− 0.03	0.57	− 0.00	1.00	0.04	0.48	0.09	0.11	0.12	*0.04*
Nocturnal sleeping duration (30 months)	0.03	0.58	− 0.01	0.88	− 0.00	0.99	0.02	0.67	0.02	0.75	0.01	0.82
Total sleeping duration (30 months)	0.05	0.39	0.05	0.37	0.08	0.17	0.04	0.50	0.02	0.73	0.02	0.73
Sleep onset time (42 months)	0.23	*< 0.01*	0.11	0.19	0.06	0.45	0.10	0.23	0.26	< 0.01	0.18	*0.02*
Nocturnal sleeping duration (42 months)	0.18	*0.02*	0.06	0.44	0.06	0.45	0.12	0.14	0.06	0.44	0.17	*0.03*
Total sleeping duration (42 months)	0.18	*0.02*	0.09	0.28	0.07	0.35	0.10	0.20	0.14	0.07	0.12	0.13

Italic values indicate significance of *P* value (*P* < 0.05)

**Fig. 1:**
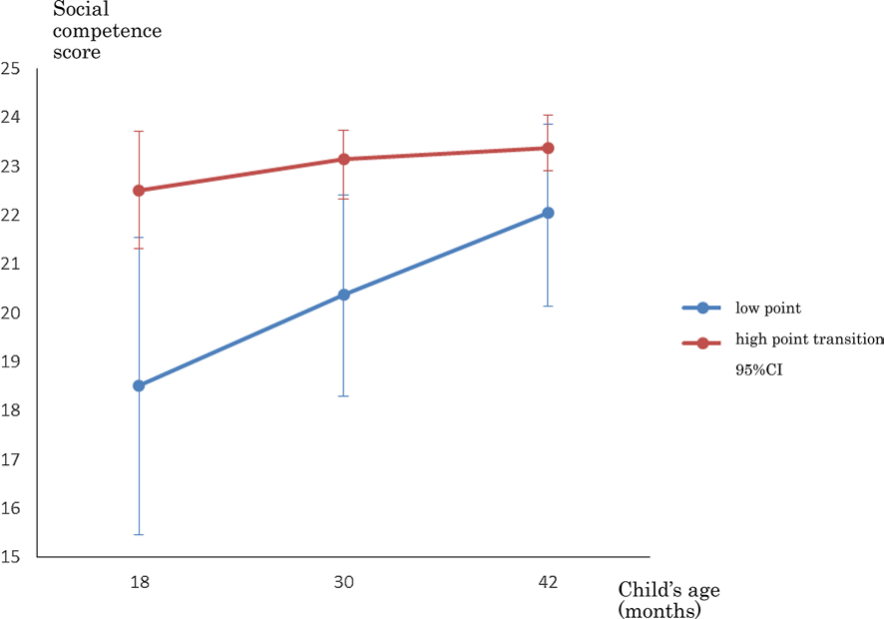
Trajectory of social competence

Figure 1 indicates the trajectory of the development of social competence from 18 to 42 months. We identified groups using a group-based trajectory model. In order to determine the optimal number of trajectories needed to describe the transition of social competence from 18 to 30 and to 42 months, we fitted models with one, two, three, four, and five profiles, based on BIC. The BIC was − 1515.28 for one trajectory, − 1517.88 for two, − 1525.27 for three, − 1528.35 for four, and − 1536.98 for five, when considering social competence alone. Using the BIC criterion, the one-group model fit the best. However, the AIC criterion in one-group was − 1509.59 and in two-group was − 1506.50, hence a two-group model was selected. The trajectory groups were divided into two groups: low point and high point transition groups. The two groups’ score came close at 42 months, but there was a significant difference between the average score of the two groups (Fig. 1).

All variables were checked in terms of which trajectory group they belonged to. Gender and the presence of siblings at 18 months emerged as significant factors. Further, “Work together with your partner to raise your child” emerged as a significant factor. Regarding sleep variables, total sleep duration and sleep onset time emerged as significant factors (Table 4).

**Table 4:** Correlation between the trajectories of children’s social competence

Items	Low vs. high analysis	
	Regression coefficient	P
Genders	2.90	*< 0.01*
Siblings	2.91	*< 0.01*
Family type	0.30	0.76
Mother’s age	− 0.55	0.58
Father’s age	− 1.02	0.31
Mother’s career	0.54	0.59
Annual family income	− 0.79	0.43
Using child care center	0.00	1.00
Play with your child	− 0.68	0.50
Read books to your child	0.97	0.33
Sing songs with your child	1.73	0.08
Work together with your partner to raise your child	1.95	*0.05*
Eat meals together as a family	0.86	0.39
Go grocery shopping with your child	− 0.20	0.85
Go to the park with your child	1.17	0.24
Go to friends’ or relatives’ house	− 0.95	0.34
Have child care support	0.67	0.50
Have consult	− 0.01	1.00
Talk with your partner about your child	0.61	0.54
When your child splits milk	0.24	0.81
Number of times in a week you slap your child	1.12	0.26
Sleep onset time	2.05	*0.04*
Nocturnal sleeping duration	1.85	0.07
Total sleeping duration	2.10	*0.04*

Italic values indicate significance of *P* value (*P* < 0.05)

We divided children into two groups, one by nocturnal sleep duration and sleep onset time, and another by total sleep duration and sleep onset time. The participants were divided into two groups with sleep onset times above the 25th percentile (before 22:00, n = 132, 63.8%; after 22:00, n = 73, 36.2%) and sleep duration below the 75th percentile (less than 10 h, n = 165, 79.7%; more than 10 h, n = 42, 20.3%). The sleep duration percentile differed from the sleep onset time percentile as we wanted to include any duration less than 11 h, given that previous literature has shown associations between sleep durations less than this amount and increased risk of high hyperactivity–impulsivity scores and low cognitive performance [21]. The sleep duration variable was therefore set at the 75th percentile. For the same reason, the participants were divided into two groups with total sleep duration below the 75th percentile (less than 12.25 h, n = 160, 77.3%; more than 12.25 h, n = 47, 22.7%). Sleep onset time was found to be significantly positively correlated with nocturnal and total sleep duration (r = 0.40, P < 0.01; r = 0.27, P = 0.04).

Using these groups, we created four new variables (X) denoting nocturnal duration and sleep onset time: (1) nocturnal sleep duration more than 10 h and sleep onset time before 22:00 (n = 68); (2) nocturnal sleep duration more than 10 h and sleep onset time after 22:00 (n = 7); (3) nocturnal sleep duration less than 10 h and sleep onset time before 22:00 (n = 97); and (4) nocturnal sleep duration less than 10 h and sleep onset time after 22:00 (n = 35; Table 5).

**Table 5:** New variables from sleeping

Sleeping variables		n	%	New variables		
Sleep onset time	Nocturnal sleeping duration			X1	X2	X3
0	0	68	32.9	0	0	0
0	1	7	3.4	1	0	0
1	0	97	46.9	0	1	0
1	1	35	16.9	0	0	1

Sleeping variables		n	%	New variables		
Sleep onset time	Total sleeping duration			Y1	Y2	Y3
0	0	64	30.9	0	0	0
0	1	11	5.3	1	0	0
1	0	96	46.4	0	1	0
1	1	36	17.4	0	0	1

An additional four variables (Y) denoting total sleep duration and sleep onset time were also created: (1) total sleep duration more than 12.25 h and sleep onset time before 22:00 (n = 64); (2) total sleep duration more than 12.25 h and sleep onset time after 22:00 (n = 11); (3) total sleep duration less than 12.25 h and sleep onset time before 22:00 (n = 96); and (4) total sleep duration less than 12.25 h and sleep onset time after 22:00 (n = 36; Table 5).

In the multinomial logistic regression analysis, we examined the relative contributions of two parenting practices at 18 months: “Work together with your partner to raise your child,” and the caregiver slapping their child or not, because there are reports that slapping the child is correlated to social competence [65–68]. The child’s gender and the presence of siblings at 18 months were entered as covariates in order to control for these effects on child social competence development. The results indicated that children with high social competence trajectories were more likely to have an earlier sleep onset time and sleep longer at night (regression coefficient = 2.15, P = 0.03; Table 6).

**Table 6:** Multinomial logistic regression analysis of sleeping new variable X and trajectory of social competence

Items	Low vs. high multiple analysis	
	Regression coefficient	P
Genders	2.35	*0.02*
Siblings	1.23	0.22
Work together with your partner to raise your child	1.69	0.09
The caregiver slapping their child or not	0.92	0.36
New variableX1 [sleep onset time, nocturnal sleeping duration = 1,0]	0.50	0.62
New variableX2 [sleep onset time, nocturnal sleeping duration = 0,1]	1.42	0.16
New variableX3 [sleep onset time, nocturnal sleeping duration = 1,1]	2.15	*0.03*
Constant	− 2.00	0.05

BIC = − 1104.67

Italic values indicate significance of *P* value (*P* < 0.05)

Furthermore, children with high social competence trajectories were more likely to have an earlier sleep onset time and have more total sleep (regression coefficient = − 2.01, P = 0.05; Table 7).

**Table 7:** Multinomial logistic regression analysis of sleeping new variable Y and trajectory of social competence

Items	Low vs. high multiple analysis	
	Regression coefficient	P
Genders	1.57	0.12
Siblings	0.75	0.46
Work together with your partner to raise your child	1.62	0.11
The caregiver slapping their child or not	0.67	0.50
New variableY1 [sleep onset time, total sleeping duration=1,0]	0.82	0.41
New variableY2 [sleep onset time, total sleeping duration=0,1]	1.15	0.25
New variableY3 [sleep onset time, total sleeping duration=1,1]	2.01	*0.05*
Constant	− 1.18	0.24

BIC = − 1110.89

Italic values indicate significance of *P* value (*P* < 0.05)

## Discussion

### Relationship between sleep variables (nocturnal sleep duration, total sleep duration, and sleep onset time) and social competence

We found that sleep onset time before 22:00, sleeping at night for more than 10 h, and total sleep duration of more than 12.25 h at 18 months of age are important for the development of social competence. Late bedtimes have been associated with problematic behavior [22, 28–42], and short nocturnal sleep duration has been negatively associated with approachability and positively associated with hyperactivity–impulsivity and social engagement [21, 25, 43, 44]. Further, total sleep duration has been associated with emotional problems [45, 46]. IRS has evidence in terms of discriminant validity for pervasive development disorder (PDD), attention deficit/hyperactivity disorder (ADHD), and abused children. Children with PDD, ADHD, and abused children have been reported to have lower levels of empathy and self-control in areas such as motor regulation and emotional regulation compared to children without these conditions [58]. Though the correlation between sleep variables and IRS is weak, these reports support our findings.

Sleep onset time had a weak correlation with motor regulation and emotion regulation at 42 months. Emotion regulation is the child’s ability to adjust his or her emotional state to a comfortable and appropriate level. Motor regulation is the child’s physical focus on a given task, and high regulation is neither overactive nor underactive. As previously indicated, late bedtimes have been associated with problematic behavior [22, 28–42, 69]. One study showed that 4-year-old children whose sleep onset time was late were aggressive [69]. Further, another study showed that 3-year-old children whose sleep onset time was late had short tempers [41]. Our results extend these findings to indicate that emotion and motor regulation skills are associated with earlier sleep onset time.

Short nocturnal sleep duration has been negatively associated with approachability and positively associated with hyperactivity–impulsivity and social engagement [21, 25, 43, 44]; our results support this finding. In 42-month-olds, social competence was significantly associated with sleep onset time and sleep duration. It is interesting that 18-month-olds’ sleep patterns were more closely related to social competence at 42 months of age than at 18 or 30 months of age. As noted previously, infant sleep patterns may affect behavior at older ages [21, 33, 69]. Children who slept less than 11 h before they were 3 years old were found to show hyperactivity–impulsivity at 6 years of age [43]. Further, children for whom parents felt their child’s sleep time was short showed aggressive behavior 14 years later [70]. Thus, infant sleep may affect social competence later in life.

Total sleep duration and sleep onset time influenced the trajectory of the development of social competence in children. Total sleep duration appears to be associated with social-emotional problems [38]. Further, reports have indicated that children with short nocturnal sleep duration are at an increased risk of high hyperactivity–impulsivity and low cognitive performance [21]. Furthermore, a significant association between sleep onset time at 3 years of age and quality of life in the first year of junior high school has been reported [71]. Our results therefore indicate that sleep onset time and sleep duration are important to the development of social competence.

Many other sleep patterns require further examination. Research has reported that late bedtimes are specifically associated with shorter nocturnal sleep duration [21]. In this study, a relationship was found between bedtimes and sleep duration (nocturnal and total sleep). Hence, examining only one of these factors may be insufficient. It is therefore necessary to examine sleep duration and sleep onset time together.

### Participants’ demographic data, sleep variables, and social competence

Participants’ demographic data indicated that they accurately represented the general population of Japan. The mean (SD) sleep onset time in this study was somewhat later than in the paper by Kohyama et al. [72], which was 21.26 (1.01). However, the means of nocturnal and total sleep duration were similar to results reported by Kohyama [65], which were 9.52 (0.94) h and 11.65 (1.27) h, respectively. The children who participated in Kohyama’s study [72] were aged 12–23 months; as the children were 18 months old in our study, age may account for some of this difference.

We chose 18 months as the age to begin measuring sleep because sleep patterns vary in the first few years of life, and it is easier to compare sleep patterns when children exhibit a given pattern consistently. Most children sleep through the night by 18 months and, while some children may continue to take two or more naps, most children take only one nap [73]; we therefore examined children aged 18 months old. Further, in Japan, infant health checkups are scheduled at 3, 6, 9, 18, and 36 months, which facilitated our assessments.

Japanese children have the shortest total sleep duration of 18 examined countries [47]; total sleep duration in this study was close to the amount reported previously [47]. Touchette et al. argue that nocturnal sleep duration of 11 h or more before age 3.5 is necessary for unimpaired cognitive performance at age six [21]. In the present study, only 7.1% of children were sleeping more than 11 h at night; hence, Japanese children may not be sleeping enough. They also fall asleep late: 30% of them go to sleep after 22:00 [49]. In this study, average sleep onset time was close to 22:00, and 36.3% of children went to sleep later than 22:00.

The mean score on the IRS was over 20 for every age group, showing that 70–80% of children of various ages have a score of above 20 [74]. This scale assesses the interaction between child and caregiver; as noted previously, the interactions that occur between child and caregiver are important to the development of social competence in the child [9]. The caregivers who participated in this JST project were interested in learning about child rearing, which may have had some impact on the high scores that were observed.

Social competence correlates with many other factors, including gender, birth order [75], family dynamics [76], and family background [77]. Identifying the most important associated factors is necessary to achieve the greatest insight and understanding of the role of sleep in later development. In this study, the trajectory of the development of social competence was related to gender and presence of siblings at 18 months. The trajectory of social competence was not related to ICCE scores; however, there was a trend toward a positive relationship between social competence and co-parenting at 18 months. We therefore included these three variables—gender, siblings, and co-parenting—in our analysis. Studies have also reported that punishment affects social competence [65–68]; we therefore used this variable too.

Regarding social competence, between low point and high point transition groups, the point came closer at 42 months. Social competence is known to develop through training [78]. It keeps developing, but unfortunately, the total score of IRS is 25 points. If IRS had higher points, the high point transition group may have had higher points at 42 months, and there could have been more difference between the two groups. To resolve this problem, we created another scale called the Interaction Rating Scale between Children (IRSC) [79]. Further investigation is needed to find differences between the two groups using the IRSC.

### Limitations and suggestions for future research

This study has certain limitations. First, we examined children only at ages 18, 30, and 42 months; future studies should use assessments that are more frequent and study older children. Second, the children’s caregivers provided information regarding sleep variables; however, the JCSSQ has verified the reliability of parent reports [52, 53]. Third, we examined sleep patterns only at 18 months of age. Future research could expand this study’s results by including older children and measuring sleep patterns at different ages.

Sleep onset time and sleep duration affect the development of social competence. The present results indicate that Japanese children’s sleep duration is short and sleep onset time is late. As mentioned previously, infant sleep may affect children later in life. There are reports that show associations between late sleep onset time at 3 years of age and low quality of life in the first year of junior high school [71]. Japanese children’s short sleep duration and late sleep onset time may have some association with social competence problems in later years. Further investigation of the role of sleep in the development of social competence is necessary. This information may become a protective factor in preventing childhood problems.

## Conclusions

We examined the association between sleep and the development of social competence in infants. Nocturnal sleep duration, total sleep duration, and sleep onset time had positive correlations with children’s social competence. Sleep is an important factor in the development of children’s social competence. Follow-up studies are necessary to investigate the role of sleep in social competence.

## Abbreviations

JST: Japan Science and Technology Agency
IRS: Interaction Rating Scale
ICCE: Index of Child Care Environment
JCSSQ: Japan Children’s Study Sleep Questionnaire
HOME: Home Observation for Measurement of the Environment
